# Seroepidemiology of *Strongyloides stercoralis* amongst immunocompromised patients in Southwest Iran

**DOI:** 10.1016/j.parepi.2016.08.001

**Published:** 2016-08-05

**Authors:** Reza Rafiei, Abdollah Rafiei, Mahmoud Rahdar, Bijan Keikhaie

**Affiliations:** a Department of Parasitology, Faculty of Medicine, Jundishapur University of Medical Sciences, Ahvaz, Iran; b Department of Parasitology, Faculty of Medicine, Infectious and Tropical Diseases Research Center, Health Research Institute, Ahvaz Jundishapur University of Medical Sciences, Ahvaz, Iran; c Department of Oncology, Shafa hospital, and Health Institute, Research Center of Thalassemia and Hemoglobinopathies, Ahvaz Jundishapur University of Medical Sciences, Ahvaz, Iran

**Keywords:** *Strongyloides stercoralis*, Nematode, Strongyloidiasis, Iran

## Abstract

Strongyloidiasis is a life-threatening parasitic infection, especially in immunosuppressed patients, with death often occurring within several days. The disease has a worldwide distribution and is endemic in tropical and subtropical regions. Therefore, this study was conducted to investigate seroepidemiology of *Strongyloides* infection amongst immunocompromised patients in Southwest Iran. This cross-sectional study was conducted amongst a population of immunocompromised patients who were referred to health care or hospital referral centres in Ahvaz, Southwest Iran. Serum samples were tested by an enzyme immunoassay for *anti*-IgG *Strongyloides* antibody. Anti-*Strongyloides stercoralis* antibody was detected in 39 of 270 immunocommpromised cases, yielding a prevalence of 14.4%. No significant differences were indicated in terms of gender, age, or type of immunocompromised disorder with *anti*-*Strongyloides stercoralis* antibody levels. In conclusion, our results demonstrated high seroepidemiology of infection with this parasite in the region. Therefore, it appears immunocompromised patients should be tested for this infection using sensitive tests. However, current research underscores that strongyloidosis must not be neglected, and further assessments in high risk population are warranted.

## 1 Introduction

Strongyloidiasis is a condition caused by *Strongyloides stercoralis*, which may be established in humans for extended periods of time because of its auto-infective life cycle. It may become life-threatening especially in immunocompromised hosts (Grove, 1996, Sharifdini et al., 2014). Strongyloidiasis symptoms may vary from subclinical in acute and chronic infection to severe and fatal in hyper infection syndrome and disseminated strongyloidiasis which, if untreated, may approach mortality rates of up to 90% (Fernando et al., 2014, Annette et al., 2009).

The disease has worldwide distribution and is endemic to tropical and subtropical regions, such as Latin America, Asia, parts of Africa and the southeastern United States (Genta, 1989, Steinmann et al., 2007, Scha et al., 2013, Román-sánchez et al., 2003). Strongyloidiasis has also been reported in most parts of Iran with a higher prevalence amongst immuocompromised populations (Zali et al., 2004, Nilforoushan et al., 2007, Meamar et al., 2007a, Froutan et al., 2008, Keiser and Nutman, 2004).

Recent developments in medical care have resulted in increasing numbers of immunocompromised individuals living for longer periods, which increases their risk of exposure for a longer duration, (Roseman et al., 2013, Paul et al., 2010). Parasitological diagnosis is often time-consuming, as *Strongyloides* larvae in the stool are frequently absent or present in very small numbers (Afzal et al., 2001). Recently, serological assays have been demonstrated to have reliable sensitivity and specificity for antibody detection (Salvador et al., 2014, Bisoffi et al., 2014, Lindo et al., 1995). Thus, the current study was conducted to investigate the seroepidemiology of *Strongyloides* infection amongst immunocompromised patients in Southwest Iran.

## 2 Materials and methods

### 2.1 Study population and sample collection

This cross-sectional study was carried out amongst a population of immunocompromised patients referred to health care or hospital referral centres in Ahvaz, Southwest Iran. Blood samples were collected from participants from March–July 2015. Participants suffered from leukaemia, HIV infections and different types of cancer. Demographic information including gender, age and immunocompromised disorders were collected from hospital and medical official documents. All blood specimens were centrifuged, and the serum separated and frozen at − 20 °C prior to analysis for parasite-specific IgG antibodies pending examination by an enzyme-linked immunosorbent assay (ELISA) for *S. stercoralis*.

### 2.2 Serology assay

An enzyme immunoassay for the diagnosis of human strongyloidiasis (Bordier affinity products SA, Switzerland) was used. The method was carried out according to the manufacturer's instructions. Briefly, wells pre-coated and sensitised with *Strongyloides* somatic larval antigens were blocked with PBS-Tween for 15 min. Wells were washed with PBS-Tween then diluted sera [1:200] were added and incubated at 37 °C for 15′. Serum was removed and the plates were washed four times. Alkaline phosphatase was added, plates washed, followed by the substrate. Finally, the reaction was stopped using K_3_PO_4_. Well absorbances were measured at 405 nm. The cutoff point was used to discriminate optimally between the sera of clinically documented strongyloidiasis cases and normal human sera. The ELISA had a sensitivity of 88% and a specificity of 94%. In addition to positive and negative controls from manufacturer using a local strongyloidiasis serum, readings were confirmed by stool examination, which was also included in each run of ELISA assay.

## 3 Results

The study population included 166 (61.5%) AIDS-defining illness cases, 36 (13.3%) leukaemia cases and 68 (25.2%) other cancer cases. *S. stercoralis* antibody was detected in 39 immunocommpromised individuals, yielding a prevalence of 14.4%. The frequency of seropositivity and immunosupresive characteristics of the study samples are shown in Table 1. As indicated, the highest rate of antibody detection (15.7%) was found in HIV^+^ cases.

**Table 1:** Frequency of seropositivity of *S. stercoralis* infection amongst immunocompromised patients, Southwest Iran.

Immunocompromised cases	Number	IgG positive	
		No	Frequency
HIV	166	26	15.7%
Leukaemia	36	4	11.1%
Cancer	68	9	13.2%
Total	270	39	14.4%

No significant differences were recorded in terms of gender, age, or immunosuppressive disorder with *anti*-*S. stercoralis* antibody levels (Table 2).

**Table 2:** Age distribution and laboratory findings of *S. stercoralis* infection amongst immunocompromised patients, Southwest Iran.

Age (Year)	Number	Frequency	IgG positive	
			Number	Frequency
0–20	8	3%	2	25%
21–40	146	54.1%	22	15%
41–60	79	29.2%	10	12.6%
> 60	37	13.7%	5	13.5%
Total	270	100%	39	14.4%

Additionally, the gender distribution of seropositivite females and males has been reported to be 16.7% and 13.3%, respectively (Table 3).

**Table 3:** Gender distribution and laboratory findings of *S. stercoralis* infection amongst immunocompromised patients, Southwest Iran.

Gender	Number	Frequency	IgG positive	
			Number	Frequency
Female	90	33.3%	15	16.4%
Male	180	66.7%	24	13.3%
Total	270	100%	39	14.4%

## 4 Discussion

Strongyloidiasis is a parasitic infection characterised by persistent infection, dissemination and the development of potentially fatal disease. This infection has a worldwide distribution, although no precise estimate is available. The present study shows the seroepidemiological prevalence of *S. stercoralis* infection amongst immunocompromised cases in Southwest Iran to be 14.4%. Meamar et al. conducted an investigation on strongyloidiasis in 781 HIV^+^/AIDS patients by the stool examination technique. According to their results, two were determined to be infected (Meamar et al., 2007b). One report showed a 4.9% *S. stercoralis* infection rate amongst rural area of Mazandaran province in Iran during 2007 (Kia et al., 2007). Additional research indicated, 42% of a population with eosinophilia was positive for *S. stercoralis* in Gilan province (Ashrafi et al., 2010). In an institution for the mentally disabled in southern Iran, 17.3% of the residents were found to be infected with this nematode (Shokri et al., 2012).

Our results are in agreement with other studies (Kia et al., 2007, Ashrafi et al., 2010, Shokri et al., 2012), regarding the high seroprevalence of *S. stercoralis* infection in immunocompromised patients. Although, the prevalence rate of this parasite in Iran is unclear, all previous studies have demonstrated that strongyloidiasis is still prevalent in some parts of the country, which are known as endemic areas (Meamar et al., 2007b, Nesheli et al., 2011, Kia et al., 2008, Tabei et al., 2009).

Most previous studies are in agreement that the prevalence of this disease is largely underestimated (Montes et al., 2010). These data are mostly based on surveys aimed at defining the prevalence of parasitic infections, without using adequate diagnostic techniques for *S. stercoralis*. To the best of our knowledge, all the previous Iranian studies used different stool examination methods as their diagnostic, which is not sensitive, especially for chronic and asymptomatic cases (Paul et al., 2010, Bisoffi et al., 2014, Bisoffi et al., 2013). In a stool- and sero-survey for *S. stercoralis* conducted in a community in the Peruvian Amazon region, the parasite was identified in the stools of 8.7% of cases and in the sera of 72% of the cases by ELISA. Lindo et al. reported a prevalence of 3.5% by stool examination and 24.2% by ELISA in Jamaica (Lindo et al., 1995). These results confirm the higher sensitivity of serological tests verses stool examinations. The ELISA has served as an excellent screening test for strongyloidiasis (Yori et al., 2006, Zueter et al., 2014, Glinz et al., 2012). Despite the high prevalence of this infection, an understanding of its epidemiology and seroepidemiology is limited (Requena-Me'ndez et al., 2013, Naidu et al., 2013, Mounsey et al., 2014).

No significant correlation was observed in terms of *S. stercoralis* infection and age in the current study. Some researchers reported increasing prevalence with age, but there is no clear explanation for this finding (Lindo et al., 1995). Our data are in agreement with most previous studies, which suggest that exposure to the parasite may not be dependent on age or gender (Lindo et al., 1995).

The current study showed a higher rate of infection in AIDS patients, although without a statistically significant correlation. Although all patients were immunocompromised, this finding may be due to the low socioeconomic status of the community.

In conclusion, our results indicate high seroepidemiology of *S. stercoralis* infection in several risk groups in Southwest Iran. To prevent the fatal consequences of this lethal nematode, immunocompromised patients should be tested for this parasite using sensitive tests. Although adequate information on the prevalence of this disease is still lacking from many parts of the country, current research suggests that *S. stercoralis* must not be neglected.

## Conflict of interest statement

We declare that we have no conflict of interest.
